# Perceived Empathy of Service Providers Mediates the Association between Perceived Discrimination and Behavioral Intention to Take Up HIV Antibody Testing Again among Men Who Have Sex with Men

**DOI:** 10.1371/journal.pone.0117376

**Published:** 2015-02-18

**Authors:** Jing Gu, Joseph T. F. Lau, Zixin Wang, Anise M. S. Wu, Xuhui Tan

**Affiliations:** 1 School of Public Health, Sun Yat-sen University, Guangzhou, China; 2 Centre for Health Behaviors Research, School of Public Health and Primary Care, Faculty of Medicine, The Chinese University of Hong Kong, Hong Kong, China; 3 Shenzhen Research Institute of the Chinese University of Hong Kong, Shenzhen, China; 4 Department of Psychology, Faculty of Social Sciences, University of Macau, Macao, China; 5 School of Public Health and Tropical Medicine, Southern Medical University, Guangzhou, China; David Geffen School of Medicine at UCLA, UNITED STATES

## Abstract

HIV antibody testing is a key measure of HIV prevention for men who have sex with men (MSM). The World Health Organization recommends sexually active and at-risk MSM to take up HIV antibody testing regularly. This study aimed to investigate the prevalence of behavioral intention to take up HIV antibody testing in the next six months among Hong Kong MSM who were ever-testers. An anonymous cross-sectional survey recruited 326 MSM who had taken up HIV antibody testing from gay-friendly venues and internet in Hong Kong. Of the participants, 40.8% had had unprotected anal intercourse with regular or non-regular male sex partners in the last six months; they were at risk of HIV transmission despite experience in HIV antibody testing. Only 37.2% showed a strong intention to take up HIV antibody testing again in the next six months. Adjusted analysis showed that both perceived discrimination toward Hong Kong MSM (AOR = .60, 95% CI: .36–.98) and the CARE Measure assessing perceived empathy of service providers (AOR = 1.05, 95% CI: 1.02–1.08) were significantly associated with intention for retesting. Perceived discrimination, however, became statistically non-significant (AOR = .68, 95% CI: .41–1.14), when both CARE Measure and perceived discrimination entered into the adjusted model. It is warranted to increase HIV retesting rate by removing perceived discrimination and reducing the negative effect of perceived discrimination through enhancement of empathy of service providers.

## Introduction

HIV is a serious epidemic among men who have sex with men (MSM) in China. In this key population, previous studies showed that the HIV prevalence exceeded 20% in a number of cities [1] and the HIV incidence was about five per 100 person-years [2,3]. In Hong Kong, the HIV prevalence was 4.31% in 2008 [4]. As a global strategy for the control of HIV, the World Health Organization (WHO) recommends health workers to increase case detection by promoting HIV antibody testing [5]. Previous studies have shown that HIV voluntary counseling and testing (VCT) is effective for both case detection and reduction of HIV-related risk behaviors [6,7]. It has been used a key measure of HIV prevention targeting MSM populations in China [8]. However, despite availability of free VCT services, the prevalence of life-time HIV antibody testing among MSM in mainland China ranged only from 18% to 47.9%, while it was 56.5% in Hong Kong in 2008 [9–12]. The proportion of MSM in China knowing about their HIV status (26%) is hence lower than those of the U.S. (56%) and Europe (73.7%) [13–16].

Unprotected anal sex (UAI) is prevalent among MSM [17,18], even among those who have ever taken up HIV antibody testing [12,19,20]; such ever-testers remain at high risk of HIV transmission. Case detection for ever-testers through regular HIV antibody testing is especially important as recent evidences show that with good adherence, the provision of earlier anti-retroviral therapy (ART) to HIV-infected individuals can reduce the risk of HIV transmission through sexual intercourse by up to 96% [21]. This effective treatment as prevention (TasP) approach, however, requires an efficient care cascade, which starts with HIV antibody testing and ends up with viral load suppression [22]. Low HIV antibody testing rate would largely diminish the potential effectiveness of using ART for prevention. The WHO guideline recommends MSM with high risk behaviors to take up HIV testing every six to twelve months [23], while the U. S. CDC recommends sexually active MSM to take up HIV testing every three to six months, in order to detect HIV infection and prevent ongoing transmission [24]. There is a dearth of studies investigating how well such guidelines have been followed by MSM.

A number of studies have identified factors associated with previous HIV antibody testing [12,20,25]. However, such studies included participants who had taken up HIV antibody testing only once or multiple times. It hence cannot inform health care workers on how to increase the likelihood of MSM returning for another HIV antibody testing in the future, as factors associated with HIV antibody testing in the past among all MSM may be different from those associated with intention to take up HIV antibody testing again in the future among ever-testers. Furthermore, according to the Trans-theoretical Model, an individual needs to go through five stages of change (pre-contemplation, contemplation, preparation, action and maintenance) before adopting a health-related behavior in the long term [26,27]. Those who had taken up HIV antibody testing only once are in the action stage, whilst those who had taken up HIV antibody testing multiple times are in the maintenance stage. It has been shown that different health promotion strategies are required for individuals at different stages of change [26–28]. Maintenance theory suggests that experience and satisfaction in performing a behavior are strong determinants of maintaining such a behavior [29]. Perceived discrimination exhibited by service providers is certainly a negative experience, while perceived empathy of service providers administering the HIV antibody testing is a satisfying experience of VCT among MSM. It is therefore warranted to understand associations between these two variables and behavioral intention for HIV retesting and their inter-relationship, as such factors are modifiable through interventions.

Perceived discrimination toward MSM is a hurdle against adoption of VCT among MSM [30]. As the Chinese culture does not approve of same-sex sexual behaviors among men [31,32], it is common for Chinese MSM to perceive discrimination against people like themselves [32,33], which has shown to be inversely associated with past HIV antibody testing behavior [12]. Furthermore, there are numerous cases reported that health service providers discriminated against MSM during their service utilization [34]; such acts would further increase perceived discrimination and hinder promotion of regular HIV antibody testing [35]. Perceived discrimination/stigma toward MSM is potentially modifiable [36]. It is warranted to reduce perceived discrimination, and to understand the mechanism (mediator) operating between perceived discrimination and behavioral intention for HIV retesting among MSM.

Perceived quality of service providers has found to be significantly associated with past HIV antibody testing behavior [25]. Empathy (defined as the ability to communicate an understanding of the user’s world and to act on that understanding in the clinical context) of health service providers is one of such important qualities [37,38]. It is associated with various types of health service utilization, such as specialty care, pain assessment and treatment, mental health service, services for child welfare and at-risk youths [39]. To our knowledge, there is however, no study investigating significance of association between perceived empathy of service providers and HIV antibody testing among MSM.

**Fig 1:**
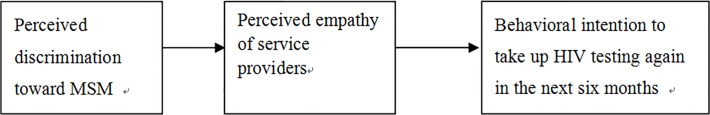
Mediating effect of perceived empathy of service providers of the association between perceived discrimination toward MSM and behavioral intention to take up VCT in the next six months.

In contrast to discrimination, empathy shown by service providers, which is both cognitive and affectionate in nature, involves effective communication between the service provider and the client [40]. Such communication shares awareness, build up shared feelings and ultimately rapport, and requires an accurate understanding and acceptance of the clients’ feeling and expectation, professional integrity and warmth [41]. Previous studies have shown that perceived discrimination causes significant damages to effective communication between health service providers and their clients [42]. Empathy is also negatively associated with discrimination [42]. Therefore, MSM who perceived discrimination would be less likely than others to perceive empathy from health service providers. As empathy is a part of the communication process between health service providers and their clients [40], there are strong reasons to believe that there exists a mechanism that perceived discrimination would diminish perceived empathy, and in turn reduce behavioral intention to take up VCT among VCT ever-testers who are MSM. This mediation hypothesis was tested statistically in the present study (see Fig. 1). It is possible to increase empathy through training. If perceived empathy is shown to be a mediator, an increase in service providers’ empathy would be a potential means to alleviate negative impact of perceived discrimination onto maintenance of VCT behavior among MSM. It is also possible that perceived empathy of service providers moderates the association between perceived discrimination and behavioral intention for HIV retesting among MSM. Perceived empathy would then have additional protective effect in alleviating the magnitude of the negative effect of perceived discrimination onto HIV retesting. Also, such a moderation hypothesis has never been tested.

This study hence investigated the prevalence of behavioral intention to take up HIV antibody testing in the next six months among Hong Kong MSM who were ever-testers. Behavioral intention is a strong predictor of future behavior according to literature [43–45] and it has been used in many HIV-related studies as the main outcome variable [17,46]. In addition, we investigated three types of associated factors of behavioral intention to take up HIV antibody testing in the next six months among MSM who were ever-testers, including: 1) background factors (e.g., education level, HIV-related knowledge, UAI with men), 2) perceived discrimination toward MSM in Hong Kong, and 3) perceived empathy of service providers administering the HIV antibody testing.

We hypothesized that ever-testers with higher education level, better HIV-related knowledge, UAI with men, low perceived discrimination toward MSM in Hong Kong, and high perceived empathy of service providers would be more likely than other ever-testers to show behavioral intention to take up HIV testing again in the next six months. Moreover, we tested the hypotheses that perceived empathy of service providers would mediate or moderate the association between perceived discrimination toward MSM in Hong Kong and behavioral intention to take up HIV antibody testing in the next six months.

## Methods

### Study population and sampling

Inclusion criteria of this study were: 1) Chinese men possessing HK identification card, 2) currently and normally living in Hong Kong, 3) at least 18 years old, 4) self-reported having had anal or oral sex with men in the last six months and 5) having had ever taken up VCT in the past. The data presented in this report was based on a sub-sample (n = 326) of the overall sample of a published study (n = 577) [12], which was conducted from July through October, 2008. Another paper has also been published based on another sub-sample of the same study, including those who had never taken up VCT and looked at their behavioral intention to do so in the next six months [19]. A mapping exercise was performed by the Department of Health in 2006, identifying 21 gay-venues in Hong Kong [47]. Using computer-generated random numbers, the study involved four gay bars and four saunas where peer MSM fieldworkers approached prospective participants. Verbal informed consent was obtained before commencing the anonymous face-to-face interviews in private settings (n = 235). Written consent was not obtained in order to maintain anonymity. Instead, the interviewers signed a form pledging that they have explained the detail of the study to the participants, a practice which has been used in many other studies [48–49]. Upon completion of the interview, the participant was given HK$ 50 (about US$ 6) to compensate the time spent (about 25 minutes). In addition, the survey was publicized via five local gay websites, inviting additional eligible participants fulfilling the inclusion criteria to complete the same questionnaire online (n = 91, 27.9%). Informed consent for such participants was implied by returning the online questionnaire and no incentive was provided. The combined sampling methods have been used in some published studies [12]. The study was reviewed and approved by the Survey and Behavioural Research Ethics Committee of the Chinese University of Hong Kong.

### Measures

Background information on socio-demographic data (age, education level, current employment status, current marital status, Hong Kong residential status) was collected. Three items of knowledge on HIV/STD were asked, including “HIV infection is asymptomatic”, “HIV antibody could not be detected within one week after infection took place” and “If a person was infected with one type of STD, he/she could not be infected with other types of STD”. An indicator variable was constructed by counting the number of appropriate responses provided to the three items.

Participants were asked whether they had had UAI with male regular sex partners (RP) and non-regular sex partners (NRP) in the last six months. RP were defined as participants’ boyfriends or lovers while NRP was defined as those who were not RP [50]. The setting of the participants’ last episode of HIV antibody testing was recorded, including governmental clinics, non-governmental organizations (NGO) and other settings (private clinics, private laboratories, and self-test kits).

Participants were asked about their behavioral intention to take up HIV antibody testing in the next six months; response categories included “no chance”, “low chance”, “high chance” and “absolutely”. A binary variable was created by combining the four responses into two categories (“no”/“low chance” versus “high chance”/“absolutely”).

Participants’ perceived discrimination toward MSM was assessed by an item “Do you feel that MSM are being discriminated by the public in Hong Kong?” The answers included “not at all”, “little”, “noticeable” and “strong”, and the variable was dichotomized in data analysis (“not at all”/“little” versus “noticeable”/“strong”).

Perceived empathy of service providers in general was measured by the 10-item CARE Measure, which assesses participants’ perception on relational empathy and communication in consultation [51–52]. The scale was originally used to assess doctors’ empathy toward patients during treatment, with response options from poor (1) to excellent (5). The tool was validated in primary care settings in Hong Kong and exhibited good psychometric properties (Cronbach's alpha = .962; factor analysis showed a single solution with high item loadings ranged from .821 to .891) [53]. In this study, exploratory factor analysis also identified one single factor, which explained 72.8% of the total variance and yielded a Cronbach’s alpha of .957.

### Statistical analysis

Descriptive statistics were summarized. Univariate odds ratios and respective 95% confidence interval (CI) were reported for the associations between all background variables and the outcome (intention to take up HIV antibody testing again in the next six months). To test the hypotheses that perceived discrimination toward MSM (Model 1) and perceived empathy of service providers (Model 2) were associated with the outcome, adjusted odds ratios (AOR) and 95% CI were reported, adjusting for background variables with p<.2. In Model 3, both perceived discrimination toward MSM and perceived empathy of service providers entered the model after adjusting for background variables with p<.2. The medication effect was tested by going through three steps: 1) showing that perceived discrimination toward MSM was significantly associated with intention for HIV retesting,; 2) showing that perceived discrimination toward MSM was significantly associated with perceived empathy of service providers (the potential mediator) and 3) showing that association between perceived discrimination and intention for HIV retesting would become statistically non-significant when perceived discrimination was added to the model containing perceived empathy [54]. To test the moderation hypothesis, an interaction term was created (perceived discrimination toward MSM * perceived empathy of service providers) and its statistical significance was tested by fitting a multiple logistic regression model containing variables of the main and interaction effects. SPSS 16.0 was used for data analyses and p<.05 was taken as statistically significant.

## Results

### Background characteristics

Of the participants, 25% were 25 years old or younger (mean = 30.0 years, standard deviation (SD) = 8.5 years); 67.2% had attained at least senior high education; 73.9% were full-time employers; 93.5% were currently single and 94.1% were Hong Kong residents; 65.6% gave appropriate responses to all the three items on HIV-related knowledge (Table 1). Of those with RP (n = 166), NRP (n = 167) and either RP or NRP (n = 266), respectively 59.0%, 32.3% and 49.2% had UAI with such male sex partners.

**Table 1:** Profiles of all participants (MSM who are ever-testers, n = 326).

	n	%
***Socio-demographic characteristics***		
Mode of recruitment		
Venue-based	235	72.1
Internet-based	91	27.9
Age group		
≤25	109	34.0
>25	212	66.0
Highest education level attained		
Senior high or below	106	32.8
Above senior high	217	67.2
Current employment status		
Employed full time	238	73.9
Student	32	9.9
Other (part-time employed, not employed, resigned)	52	16.2
Current marital status		
Single	300	93.5
Other (married, divorced, etc.)	21	6.5
Residential status (possession of HK identification card)		
Non-local	19	5.9
Local	303	94.1
***HIV/STD knowledge (% with appropriate responses)***		
HIV infection is asymptomatic	276	85.4
HIV antibody could not be detected within one week after infection took place	246	76.2
If a person was infected with one type of STD, he/she could not be infected with other types of STD	295	91.3
Number of appropriate response (0–3)		
0–2	111	34.4
3	212	65.6
***Settings of the latest episode of HIV antibody testing***		
NGO	138	42.7
Governmental clinics	101	31.0
Other settings (private clinics, private laboratories, and self-testing)	93	26.3
***Timing of the last episode of HIV antibody testing***		
Less than 6 months ago	138	43.4
6–12 months ago	86	27.0
13–24 months ago	61	19.2
More than 24 months ago	33	10.4
***Unprotected anal intercourse (UAI) in the last six months***		
Any UAI with male regular partner (RP)		
No RP	154	48.1
Yes	98	30.6
No	68	21.3
Any UAI with male non-regular partner (NRP)		
No NRP	154	48.0
Yes	54	16.8
No	113	35.2
Any UAI with male RP or NRP		
No RP or NRP	55	17.1
Yes	131	40.8
No	135	42.1
***Perceived discrimination by the public toward MSM in Hong Kong***		
No	23	7.1
Little	115	34.5
Noticeable	138	42.9
Strong	50	15.5
***Perceived empathy of service providers*** *(mean* ± *s.d.)*		31.3±9.2

### Information on HIV antibody testing

All participants were ever-testers. In the last episode of their HIV antibody testing, respectively 31%, 42.7% and 26.3% went to governmental clinics, NGO and other settings for testing (Table 1). Many of the ever-testers had taken up the test at least six month ago (more than 6 months before: 56.6%; more than 13 months before: 29.6%). Only 37.2% of all participants showed strong behavioral intention (perceived a high/absolute chance) to take up HIV antibody testing in the next six months.

### Perceived discrimination toward MSM and perceived empathy of service providers

Of the participants, 58.4% perceived that the Hong Kong public discriminated against MSM (no: 7.1%, little: 34.5%, noticeable: 42.9%, strong: 15.5%). Overall, 63.7% to 81% of the participants gave positive ratings (good, very good or excellent) to the individual items of the CARE Measure assessing perceived empathy of service providers (Table 2). We found that the mean CARE Measure score varied by the setting of testing (F = 7.86, df = 2, p<.001); the mean score of those who went to NGO for testing (33.8; SD = 9.3) was higher than those who went to governmental clinics (28.0; SD = 8.5) and those who went to other settings (30.2; SD = 8.9) for testing (p<.001 and p = .011); the latter two means (governmental clinics and other settings) were not statistically different (p = .172).

**Table 2:** Item frequency distributions of the CARE Measure.^a^

*“The service provider of the HIV antibody testing was…”*	%
Making you feel at ease	
poor/fair	21.6
good/very good/excellent	78.4
Letting you tell your story	
poor/fair	27.6
good/very good/excellent	72.4
Really listening	
poor/fair	25.4
good/very good/excellent	74.6
Being interested in you as a whole person	
poor/fair	34.8
good/very good/excellent	65.2
Fully understanding your concerns	
poor/fair	35.5
good/very good/excellent	64.5
Showing care and compassion	
poor/fair	29.7
good/very good/excellent	70.3
Being positive	
poor/fair	26.1
good/very good/excellent	73.9
Explaining things clearly	
poor/fair	19.0
good/very good/excellent	81.0
Helping you to take control	
poor/fair	21.3
good/very good/excellent	78.7
Making a plan of action with you	
poor/fair	36.3
good/very good/excellent	63.7

^a^: Cronbach's alpha = .957, 72.83% of the variance was explained by the factor (identified by exploratory factor analysis).

### Factors associated with behavioral intention to take up HIV antibody testing in the next six months

Table 3 shows that none of the background variables was significantly associated with strong behavioral intention to take up HIV antibody testing in the next six months, although two variables, current marital status (married/divorced/other marital status versus single marital status: OR = 2.44, p = .051), and UAI with NRP in the last six months (having had UAI versus absence of NRP: OR = 1.81, p = .066), showed marginal statistical significance (.05<p<.1). Non-significant independent variables with p>.2 included mode of recruitment, age, employment status, HIV-related knowledge, UAI with RP in the last six months, UAI with RP or NRP in the last six months, and the setting of the last episode of HIV antibody testing.

**Table 3:** Associations between background factors and behavioral intention to take up HIV antibody testing in the next six months.

	row %	OR_u_	(95% CI)	p value
***Socio-demographic characteristics***				
Mode of recruitment				
Venue-based	35.3	1.00		
Internet-based	41.8	1.31	(.80, 2.15)	.284
Age group				
≤25	33.3	1.00		
>25	38.6	1.26	(.77, 2.04)	.359
Highest education level attained				
Senior high or below	30.8	1.00		
Above senior high	39.8	1.49	(.95, 2.45)	.117
Current employment status				
Full-time employed	34.7	1.00		
Still student	40.6	1.28	(.60, 2.73)	.515
Other (part-time employed, not employed, resigned)	44.2	1.49	(.81, 2.74)	.210
Current marital status				
Single	35.4	1.00		
Other (married, divorced, etc.)	57.1	2.44	(1.00, 5.98)^+^	.051
Residential status (possession of HK identification card)				
Non-local	52.6	1.00		
Local	36.0	.51	(.20, 1.28)	.152
***HIV/STD knowledge***				
Number of appropriate response (0–3)				
0–2	33.0	1.00		
3	38.9	1.29	(.79, 2.10)	.306
***Settings of the latest episode of HIV antibody testing***				
NGO				
No	35.5	1.00		
Yes	39.4	1.18	(.75, 1.87)	.470
Governmental clinics				
No	36.9	1.00		
Yes	37.6	1.03	(.63, 1.67)	.906
Other settings (private clinics, private laboratories, and self-test kits)				
No	35.2	1.00		
Yes	41.9	1.33	(.81, 2.17)	.259
***UAI in the last six months***				
Any UAI with male RP				
No RP	37.3	1.00		
Yes	32.3	.80	(.47, 1.38)	.426
No	44.1	1.33	(.74, 2.38)	.336
Any UAI with male NRP				
No NRP	32.2	1.00		
Yes	46.3	1.81	(.96, 3.42)^+^	.066
No	39.3	1.36	(.82, 2.26)	.237
Any UAI with male RP or NRP				
No RP or NRP	33.6	1.00		
Yes	35.7	1.13	(.70, 2.27)	.412
No	40.0	1.35	(.80, 2.31)	.251

^*+*^: .05<*p*<.1.

Adjusted for the four background variables with p<.2, perceived discrimination toward MSM (AOR = .60, 95% CI:. 36-.98) and the CARE Measure assessing perceived empathy of service providers (AOR = 1.05, 95% CI: 1.02–1.08) were both significantly associated with strong behavioral intention to take up HIV antibody testing in the next six months (see Model 1 and 2 of Table 4).

**Table 4:** Testing associations and mediating effects of behavioral intention to take up HIV antibody testing in the next six months considering factors of perceived discrimination toward MSM and perceived empathy of service providers.

	Model 1				Model 2				Model 3			
	OR_u_	(95%CI)	OR_a_	(95%CI)	OR_u_	(95%CI)	OR_a_	(95%CI)	OR_u_	(95%CI)	OR_a_	(95%CI)
***Perceived discrimination by the public toward MSM in Hong Kong***												
No/little	1.00		1.00		N.A		N.A.			1.00	1.00	
Noticeable/strong	**.58**	**(.37,.92)** *	**.60**	**(.36,.98)** *					.65	(.40,1.08)	.68	(.41,1.14)
***Perceived empathy of service providers***	N.A.		N.A.		**1.03**	(1.00, 1.05)*	**1.05**	**(1.02,1.08)** **	**1.03**	**(1.00,1.05)** *	**1.03**	**(1.01,1.06)** *
**-2*LL***		415.09		403.65		349.02		342.12		345.35		339.02

Model 1: the independent variable is perceived discrimination toward MSM.

Model 2: the independent variable is perceived empathy of service providers.

Model 3: the independent variables are both perceived discrimination toward MSM and perceived empathy of service providers.

OR_u_: odds ratio of univariate analysis.

OR_a_: odds ratio of adjusted analysis adjusting for background variable with p<.2 in Table 3. N.A.: not included in the model.

*: *p*<.05

**: *p*<.01.

### Testing the mediation and moderation hypothesis

Perceived discrimination toward MSM and perceived empathy of service providers was significantly and negatively correlated with each other (Spearman r = -.137, p = .025). The variable of perceived discrimination toward MSM, which was significant in Model 1, lost its statistical significance in Model 3 (AOR = .68, 95% CI = .41 to 1.14), which had CARE Measure added to Model 1 and adjusted for those background variables with p<.2; the CARE Measure remained statistically significant in Model 3 (AOR = 1.03, 95% CI = 1.01 to 1.06). According to the three steps required for testing mediating effects, the association between perceived discrimination toward MSM in Hong Kong and strong behavioral intention to take up HIV antibody testing in the next six months was fully mediated by perceived empathy of service providers.

The interaction term in the multivariate logistic regression model also including the two main effects of perceived discrimination and perceived empathy of service providers was statistically non-significant (p = 0.677). The moderation hypothesis was hence not supported by our data.

## Discussion

It is seen that only about one third of all the ever-testers expressed strong behavioral intention to take up HIV antibody testing in the next six months. Furthermore, only respectively 42.9% and 18.1% of those who had taken up HIV antibody testing for at least six and 13 months intended to do so again in the next six months. Discounting the discrepancy between behavioral intention and actual future behavior, the proportion of ever-testers who would actually take up HIV antibody testing would even be less. Assuming a discount rate of 40%, only about one-fifth of all ever-testers would eventually return for another HIV antibody testing within the next six months. Such situations are far from ideal and effective health promotion is greatly warranted.

Another published paper which was based on the same data showed that only 12.7% of the MSM who were had never taken up VCT (never-testers making up 44.0% of all sampled MSM) intended to take up VCT again in the next six months [19]. It is expected that prevalence of behavioral intention to take up VCT among ever-testers (this paper) was higher than that among never testers. However, piecing the two figures of the never-testers and ever-testers together, the picture reveals that despite recommendations made by international health authorities, only a minority of the MSM in Hong Kong intended to take up VCT in the next six months. It implies that only a small fraction of the HIV positive MSM in Hong Kong has been diagnosed, and potential early HIV treatment effect recommended by the WHO in lowering HIV transmission would largely be compromised. Increasing coverage of HIV testing is hence a priority of HIV prevention work targeting MSM.

Given the high prevalence of ever-testers having had UAI with NRP and/or RP, it implies that a high proportion of such MSM were at high risk of HIV transmission. The level of risk cannot be overlooked given the noticeably high prevalence of HIV among MSM in Hong Kong (close to 5%). It is important to point out those at risk of HIV transmission (e.g. having had UAI with RP/NRP) showed similarly low behavioral intention to take up HIV antibody testing in the next six months. Again, taking into account potential discount in behavioral intention without follow-up actions, the prevalence of HIV antibody retesting among at risk MSM who were ever-testers would fall short below that recommended by the U.S. CDC (sexually active MSM: every three to six months) or by the WHO (at risk MSM; every six to 12 months) [23,24].

As expected and in corroboration with the results of other reports, we found that perceived public discrimination toward MSM in Hong Kong was prevalent. In this study, perceived discrimination was found to be strongly associated with behavioral intention to take up HIV antibody testing in the next six months. It is warranted to reduce public discrimination and public stigma toward MSM, which require both structural changes (e.g. policies and legislation) and health promotion efforts. Factors associated with public discrimination toward MSM include knowledge, negative perception [55], and moral concerns [31–32], but such factors are modifiable [34]. Previous studies have shown that interventions could be effective in reducing public stigma toward MSM and/or people living with HIV [56–59]. However, health workers should be made aware of potential worry about layered stigma as those who are tested HIV positive would have to face double perceived discrimination, against his MSM status as well his new HIV positive status [60].

Besides, perceived discrimination is associated with self-stigma [61], which is prevalent among MSM [62–63]. According to some researchers, self-stigma results in concurrence with public stigma and decrement in self-esteem [64] and it is strongly associated with psychological problems [65]. Self-stigma may therefore be resulted from perceived discrimination, and may have both direct and indirect effects onto utilization of health services [65–66], including HIV antibody testing [67]. It is warranted to reduce self-stigma among MSM and a number of effective self-stigma reduction programs based on cognitive behavioral therapy have been reported, although such studies did not target MSM [66]. Research of the relationship between self-stigma and HIV antibody testing among MSM is warranted.

It is also warranted to unlock the association between perceived discrimination and HIV antibody testing. One direction is to investigate coping responses made by MSM under situations of perceived discrimination in order to reduce negative coping [68]. Another direction, which is supported by this study’s key result, is to increase perceived empathy of HIV testing service providers. The negative effect of perceived discrimination in decreasing perceived empathy could be offset by increase in perceived empathy resulted from effective interventions. In this study, perceived empathy was strongly associated with behavioral intention for HIV antibody testing. The finding is consistent with the maintenance theory that suggested that experience and satisfaction in performing a behavior are determinants of maintenance of the behavior [29]. Although the overall situation on perceived empathy of service providers was satisfactory (as two-third or more of the participants showed good responses to the items of the CARE Measures scale), there are still rooms for improvement. For instances, our data showed that slightly more than one third of the participants felt that service providers of HIV antibody testing were not interested to see them as a whole person, did not fully understand them and did not make a plan of action with them. In literature, there are effective interventions to increase empathy among health service providers [69–70]. Since the number of service providers involved HIV testing would be relatively small, training and incentives to improve the situation is highly feasible.

It is interesting to see that the setting of HIV antibody testing (NGO, governmental and other settings) was associated with perceived empathy of service providers. We expected that the mean CARE Measure score would rank in the order of NGO, governmental and other settings. The results showed that the NGO group ranked the highest but those who visited governmental clinics and those did the testing in other settings (largely commercial settings as self-test is not common among MSM in Hong Kong) did not differ in this regard. Service providers of NGO are likely to be local peer MSM workers who would have better sensitivity for needs and culture of MSM. Improvement of governmental services is hence required. We suggest that process evaluation, including assessment of perceived empathy, to be included into regular evaluation of HIV antibody testing services offered by various institutions.

Our results further showed that perceived empathy mediated the association between perceived discrimination and HIV antibody testing as the significance of the association between perceived discrimination and HIV antibody testing disappeared after controlling for CARE Measures. Therefore, our study contributes to the field as we have identified a potential pathway of which perceived discrimination might operate to reduce behavioral intention to return for HIV antibody testing—ever-testers who felt MSM are being discriminated would perceive lower empathy of service providers and hence have lower intention for retesting. Perceived discrimination has therefore another potential negative effect of lowering perceived empathy, a topic that requires further investigation. The mediator of perceived empathy is specific and potentially modifiable [34]. An important insight on how to increase behavioral intention to return for another HIV testing by promotion of empathy therefore emerges. Intervention strategies based on improvement of empathy can be derived.

The non-significant moderation effect further clarifies the inter-relationship among perceived discrimination, perceived empathy of service providers and behavioral intention for HIV retesting. It implies that the negative impact of perceived public discrimination was the same for those perceiving and not perceiving empathy. Importance of structural intervention to remove public discrimination toward MSM is reinstated.

Return for HIV antibody testing among ever-testers is of utmost importance, as with such a low rate of retesting and hence a low rate of regular HIV antibody testing among at risk MSM, the care and treatment cascade would be highly inefficient [71], and the MSM population would not be able to obtain potential benefits derived from earlier anti-retroviral treatment and TasP approaches. Many of the HIV positive MSM in China did not know about their HIV status [72]. We contended that the low prevalence of retesting is one of the reasons why the HIV incidence among MSM in China was very high (5 to 7 per 100 person-years). To our knowledge, there is no previous study reporting prevalence of regular HIV antibody testing among MSM in Hong Kong and in mainland China.

This study has a number of limitations. First, it was based neither on probability sampling (which was not feasible) nor respondent-driven sampling methods. Since many HIV interventions targeting MSM are venue-based or internet-based [73–74], the venue-based and internet-based study design of this study has a practical implication that the promotion of HIV antibody testing may take place in such settings. Second, this is a cross-sectional study and no causal relationship could be established. Third, the outcome variable of this study is behavioral intention to take up HIV antibody testing and longitudinal studies investigating actual adoption of HIV antibody testing is warranted. Fourth, perceived discrimination toward MSM in Hong Kong was based on a single question and could not be validated. Lastly, data were self-reported and reporting bias may exist although anonymity would have reduced the bias. Reporting bias would favor intention to take up HIV antibody testing; the actual prevalence of intention to take up HIV antibody testing may hence be lower than that reported in this study. Lastly, the category of other settings for the last episode of HIV antibody testing did not distinguish commercial versus home-testing settings; prevalence of over-the-counter self-administrated HIV rapid testing (HICSRT) self-testing in Hong Kong was however, low (6% in 2010, unpublished data, n = 577).

In conclusion, performing HIV testing for once only or very infrequently, but not regularly, among at risk MSM would not effectively serve the purposes of case detection and inducement of behavioral changes. Low retesting rate is possibly one of the strongest obstacles compromising the effectiveness of existing HIV prevention among MSM. This study is among the first few ones inspecting problems of HIV antibody retesting and associated factors. We found that improvement in perceived empathy of service providers is a potentially useful strategy to counteract negative impact of perceived discrimination onto HIV antibody retesting among MSM. Randomized controlled trials to be conducted in different countries are required to establish evidence base for translation into services. Such research is urgently and greatly warranted.
